# Review of Sport-Induced Groin Injuries

**DOI:** 10.5812/traumamon.12666

**Published:** 2013-10-14

**Authors:** Parisa Sedaghati, Mohammad-Hossein Alizadeh, Elham Shirzad, Abolfazl Ardjmand

**Affiliations:** 1 Department of Physical Training and Sport Sciences, Kish International Campus, University of Tehran, Tehran, IR Iran; 2 Department of Corrective Exercises and Sports Injuries, University of Tehran, Tehran, IR Iran; 3 Physiology Research Center, Kashan University of Medical Sciences, Kashan, IR Iran

**Keywords:** Sports, Wounds and Injuries, Groin, Sports Medicine

## Abstract

**Context:**

Groin injuries are among the most common injuries co-existing with sports. The aim of this review was to outline the epidemiology and identify risk factors, as well as examine preventative and interventional measures for reducing the occurrence of this form of injury among athletes.

**Evidence Acquisition:**

An electronic, systematic search for relevant keywords, either separately or in combination was sought in the academic scientific databases.

**Results:**

Groin injuries, acute or chronic, consist of a high percentage of injuries that manifest with pain. Despite the specific tendency for injury among some sports, such injuries make up 2-5% of sport-induced injuries. There are few available reports on lower limb injuries, especially groin injuries, in Iran. Numerous factors predispose to groin injuries. A lengthy list of preventive/ treatment measures, from preliminary to sophisticated, have been proposed.

**Conclusions:**

Although using a programmed strategy designed to decrease the risk of groin injuries by taking a strategic approach to exercise may alleviate complications, in some cases the chronic nature of the injury may threaten the professional life of the athlete. More research is required to plan suitable programs for reducing the risk of this type of injury in athletes.

## 1. Context

Groin injuries are among the most prevalent lower limb injuries associated with most sport activities. In addition, groin pain followed and exacerbated by sport activities is a chief complication among athletes. Groin injuries also account for a considerable percent of professional sport injuries and cause pain. Usually in a high percent of athletes the cause of pain is multi-factorial. In cases such pain as a prodrome of acute and/or chronic injury can result in problems consistent with some chronic pain syndromes. The main goal of this review was to study the incidence of groin injuries for selected sports. Upon reviewing the literature related to groin injuries, some aspects seemed more prominent. This review mainly focuses on the general epidemiology and the relevant causes of groin injury. 

### 1.1. Epidemiology of Groin Injury

In general, groin injuries make up 2 to 5 percent of all sport-induced injuries. Adductor sprain is the usual musculoskeletal etiology of pain (1). Moreover, the resultant pain is often a frustrating problem in individuals who engage in activities involving sprinting, rapid acceleration and deceleration changes (2). Reportedly, the most prevalent groin injury in sports is the adductor strain. In soccer players, an incidence rate of 10% - 18% for groin injury has been mentioned (3, 4). It is clear that sprains and pain in the pubic region in athletes have continued to worsen by some sport activities (e.g. hockey, soccer, and American football) (3, 5-9). Besides professional athletes, sports-related injuries in this area have been reported in 5% - 9% of secondary school students (10). Epidemiologically, groin injuries comprise 10 - 18% of all soccer injuries (11). Tyler et al. (2010) reported that in winter sports nearly 10% - 11% of all injuries are sprains worldwide (12). Groin sprains also comprised 10% of all injuries in Nordic hockey professionals (13). Molsa et al. (1997) also reported that in similar sports in Finland groin sprains were responsible for 43% of all muscle sprains (14). Another similar study in Nordic countries reported that the incidence rate of groin pain was 10 - 18 cases per 100 football players (15). Giza et al. (2005) reported that during 2002, almost 9.5% of all male football players had groin sprains (8). Ekstrand and Gillquist (1983) reported 32 sprains in 180 male players (13% of all cases) during a one-year period (3). Considering the above, adductor sprains are not specific to the mentioned sports. The incidence of sprains in a hockey team was 3.2 cases per 1000 players (16, 17). Besides, in most studies the rates of injuries were highest before the game season compared to after the season (9). Despite numerous studies which have been done regarding soccer injuries worldwide, there is scarcity of available data considering the epidemiology of these injuries in Iranian football players, although it is the most popular sport in our country. Halabchi reported that 21% of all injuries in women Shotokan Karate were in the lower limb region (18). In another similar study Shadanfar reported that both in professional male and female handball players, 10.1% percent of all injuries were in the thigh, hip and groin regions (19). 

### 1.2. Risk Factors 

A large body of research has proven a relation between the muscular/skeletal flexibility and sprains in different sports (3, 9, 12, 20). In all above-mentioned studies the limitation of adductor muscle strength to some extent has been followed by muscular sprains, as well (3). Arnason et al. (2004) in a study on 306 football players identified that the risk factors included a positive history of sprains and also a decreased ROM for hip abduction (20). Ekstrand and Gillquist (1983) reported limited preseason hip ROM in football players who had frequent groin sprains (3). Despite the identification of risk factors and applying preventative approaches, the disorder persists in some sports (21). Diminished hip ROM was proposed as a predisposing factor for exercise-induced chronic groin pain (21). The most common risk factors for adductor muscle sprains include stiffness (3, 21), previous history and an imbalance of hip adductors-to abductors strength (17). 

### 1.3. Causes of Groin Injury

From the causative point of view, the range of symptoms for groin injuries of these types resulting from a variety of causes can vary from non-persistent benign acute symptoms to persistent sometimes life-threatening syndromes. The resulting local or general pain can originate from single or multiple muscular/skeletal structures (5). Pain which can be regarded as the outcome of acute or chronic injury may have manifestations identical to some chronic states (6). Hackney R G (2012) in a comprehensive review on groin pain refers to common (hip joint pathology, hernia, snapping psoas) and rare causes (stress fracture, spinal pathology, intrapelvic causes) and differential diagnosis of groin pain that are manifested by abnormal gait followed by instable pubic symphysis and hernia and the concurrent existence of more than one cause of pain that persists despite treating one or few sources of pain (Table 2) (22). Moreover, Freckleton G (2011) refers to hamstring muscle sprain as the common cause of injury in exercises that require speed and acceleration changes (23). However, based on Eirale et al., overuse injuries were considered the main cause of groin injuries (24). The causes of groin pain are multi-factorial, and 27% of cases relate two more than a single factor (24, 25). However, training each of the 3 adductor muscles has been proposed as the major etiology of pain in football players (26) and skaters (17), respectively. According to Ekstrand et al. (1999) groin injuries in most cases can lead to chronic disabilities (27, 28) in which the muscular/tendinous strain of the adductor muscles and other muscles crossing the hip region is the main cause of the groin pain (4, 29 - 31). The estimated risk rate for such injuries among male football players is near 0.81 injury per 1000 hours of exercise (25). 

## 2. Evidence Acquisition

### 2.1. Literature Review 

Through an organized search strategy relevant papers during a 14-year period (1980 - 2013) were searched in the following databases: WEB OF SCIENCE, WEB OF KNOWLEDGE, SCIENCE DIRECT, PUBMED, MEDLINE, EMBASE, PEDro and SCOPUS. Keywords searched were ‘‘groin ligament injuries’’, ‘‘groin injuries’’, ‘‘groin and epidemiology’’, ‘‘groin sprain in sports’’, ‘‘groin sprain’’ and ‘‘groin pain’’. All retrieved papers, and related review papers were searched and cited. 

### 2.2. Study Classification 

The selected papers were primarily checked for title, abstract, and keywords to see whether they qualify the required classification criteria. Each paper was required to assess the incidence/prevalence and the epidemiological issues of groin injury or the interventional measures. 

## 3. Results

We reviewed groin injuries among different sport activities with an emphasis on epidemiology, mechanisms (etiology), and risk factors, preventive and interventional measures as a practical issue for clinical application. Moreover, a relationship was found between injury occurrence and issues like the time of injury and hip adduction-to-abduction strength ratio.

We found that despite the determination of risk factors, adduction strain is considered as a common problem in groin injury that continues to occur throughout most sports. High-risk sports were pointed-out and their etiologies were also ascribed to multiple factors. It was also found that the injuries may be obviated if the risk factors are determined prior to the initiation of the season. Among the multiple factors involved some may multiply the risk of adductor sprain. Based on our review, adductor sprains were identified as the more common problem among the groin injuries and accordingly contributing factors to the development of groin pain are classified as intrinsic and extrinsic. Finally, a debate on preventive measures and interventions was found whereby a three-stage system was stated for classification of groin sprains based on the prognosis of all muscle tears occurring in groin injuries. 

## 4. Conclusions

In the present study we reviewed groin injuries among different sport activities with an emphasis on epidemiology, mechanisms (etiology), and risk factors, preventive and interventional measures as a practical issue for clinical application. Reportedly, among the most common complications of groin injury is groin pain, which affects about 10-18% of individuals, annually (15, 32, 33). Identifying the main etiology of groin pain may seem quite ambiguous due to the lengthy differential nature of diagnoses (34). Considering this problem and for discarding any ambiguity, differential diagnosis of such pain in athletes (like the one presented in Table 1) may be helpful (1, 5, 26, 35). As Table 1 indicates, a broad range of diagnoses may be associated with groin pain. Groin injuries in football competitions are about 5-13% of all muscular/skeletal injuries (3, 24). 

**Table 1.** Differential Diagnosis of Groin Pain

Disorder or Disease
**Abdominal aortic aneurysm**
**Adductor tendonitis**
**Avascular necrosis of femoral head**
**Bursitis pelvic inflammatory disease**
**Diverticular disease**
**Epididymitis**
**Herniated nucleus pulposis**
**Hydrocele**
**Inguinal or femoral hernia**
**Intra-abdominal inflammation**
**Legg-Calve'-Perthes disease**
**Lymphadenopathy**
**Muscle sprain**
**Myositis ossificans**
**Nerve entrapment**
**Osteoarthritis**
**Ovarian cyst**
**Postpartum symphysis separation**
**Prostatitis**
**Seronegative spondyloarthropathy**
**Slipped capital femoral epiphysis**
**Sportman's hernia**
**Stress fracture**
**Testicular neoplasm**
**Testicular torsion**
**Varicocele**

Previous studies have revealed a relationship between strength/flexibility and musculoskeletal sprains in different sports (3, 20). As Ibrahim's study (2007) showed, an incidence of groin injury accounted for 8% of all injury cases. In soccer players, a recurrence rate of 15-20% is expected. Most of such injuries were within the initial months of the year and may clearly demonstrate the problem of over time (25). Moreover, regarding the relationship between injury occurrence and time of injury, Tyler et al. (2001) reported decreased preseason hip adduction strength of 18% among national hockey league members, which ultimately presented groin sprains (17). Also the hip adduction-to-abduction strength ratio was different between the two groups and injured cases had weaker adduction. Among the cases with sustaining sprains, compared to the uninjured side, preseason adduction-to-abduction strength ratio was significantly lower signifying a subsequent sustained groin sprain. Although comparison showed the adduction strength in such cases has abduction strength of 86% on the non-injured site, it was only 70% on the injured side. On the other hand, a research on adductor sprains in hockey reported no relationship between the peak isometric adductor torque and the sprain (17). Contrary to the results reported by Tyler, one report showed more cases with the use of a manual dynamometer may extend the variability of strength for tests and limit the variations. Despite the determination of risk factors and applying the related interventional measures, adduction sprain as a common problem in groin injury continues to occur throughout most sports. However, the exact incidence of such injuries in sports is unknown, because in most cases the injured play in spite of mild pain and injury. Moreover, superimposed diagnoses are another important notion that may affect the incidence rate. Regarding the importance of adductor sprains, the most common risk factors may include decreased flexibility of the adductor tendon (17), its weakness (16), and previous history (16, 35). Especially in soccer, relationships have also been described between stiffness of the tendon and the occurrence of sprains (3). Tyler et al. (2001) demonstrated that the preseason weakness in adductor tendon strength and a less adductor-to-abductor ratio of less than 80% were predisposition for adductor strain (17). Emery and colleagues (2001) demonstrated that the off-season players were less likely to have a groin injury, similar to national hockey league players. In the aforementioned research, the exact risk factor was the history of a preceding adductor strain (36). Tyler et al. (2001) also made a link between preexisted injuries to reoccurring injury in four out of nine (44%) cases (17). These findings are in line with the findings of Seward et al. (1993), who indicated a 32% recurrence for groin sprains among the Australian football players (35). Considering high-risk sports, some researchers documented that these activities may include Australian football, soccer, rugby, and ice hockey, respectively (36-38). In a novel review Maffey et al., investigated the risk factors and preventive approaches for strain type injury in some exercise activities (31). Tyler (2010) stated that among the sports studied, ice hockey and soccer were especially predisposed to muscle sprains. This is firstly because such injuries have been related to multiple factors, such as a previous history, hip muscle weakness, preseason training, as well as the level of prior injury. Hence, the injuries may be prevented if the risk factors are determined prior to the initiation of each season (12). The second reason is that the athletes are involved in activities that need running, particularly with rapid changes in direction, while activities like repetitive kicking and bodily contact are at the highest risk for groin pain (2). The third reason is that one common factor in all of these sports is that all such events happen mostly in athletic groups involved in activities having some sort of lateral hitting, stroke, shearing, prompt/sudden acceleration, and or abrupt directional changes (38). Morelli's study (2005), found less abductor ROM and strength linked to a higher incidence of adductor sprains (39). Furthermore, biomechanical disorders of the lower limb (e.g. excessive pronation, leg-length difference, and the imbalance of the adjacent pelvic muscles and muscular fatigue) have also been implicated to multiply the risk of adductor strain (40). Despite the existence of clinical trials for the establishment of recent preventive measures directed to ameliorate the disorder have been proven to be effective for hockey (41), Nicholas (2002) stated that adductor strain recovered insufficiently and could chronically worsen. In such cases the affliction of any of the six adductor muscles may exist. Among the predisposed groups, ice hockey and soccer players seem particularly susceptible to adductor muscle sprains. In hockey team members almost 10% of all injuries are groin sprains (4). Another aspect of the matter is the time lost per game. Emery et al. (1999) showed that groin and abdomen injury cause about 25 missing plays for each team in every season among the hockey members (7). In these games hip injuries were the most prevalent muscle sprains seen in training and competitions. Furthermore, muscular imbalance and structure asymmetry were common in hockey due to repeated rotational force to which they are exposed. The initial diagnosis and management of imbalance and asymmetry detected in these conditions may decrease the recurrence and intensity of such disorders. According to our review, adductor sprains were more common among the groin injuries. The adductor muscles of the hip joints consist of 6 muscles; the initial action of such muscles is adducting the thigh during open chain movements and also stabilizing the lower extremities and hip structures during the closed chain type motions. The adductor longus is a commonly injured muscle in sport activities (26) The absence of any mechanical advantage in adductor longus may make it more susceptible to strain type injuries. 

### 4.1. Contributing Factors to the Development of Groin Pain

There are several factors which can predispose patients to develop groin sprain. These factors need to be assessed and corrected according to the guidance of a physiotherapist. Some of these factors which are classified as intrinsic and extrinsic and are presented in Table 2. 

**Table 2.** Contributing Factors to the Development of Groin Pain (42)

Extrinsic Factors	Intrinsic Factors
Post-injury inadequate rehabilitation	Muscle weakness
Fatigue	Inadequate muscle conditioning
Decreased fitness	Poor groin flexibility
Muscle tightness	Poor pelvic and core stability
Inadequate warm up	Ethnicity/race
Poor posture	Increasing age
Neural tightness	
Recurrent injury	

### 4.2. Preventive Measures

In 2002, analyzing the data of hockey showed that adductor sprains were twenty times more frequent during training camp as compared to regular training, signifying that de-conditioning may involve injury, and applying strengthening measures to some extent may be a preventive measure. The augmentation of the hip and lower extremities may play a key role in adductor injury prevention and recently these interventions have been proved to be effective in the prevention of groin injury in football and hockey (41, 43). In another similar research, Tyler et al. (2002) reported the result of preventive intervention in national hockey league players. These augmenting/preventive plans mainly emphasized recovery of adductor weakness (at least to 80% of its strength), and such augmentation considerably lowered the injuries (41). Sprain or tendonitis of the iliopsoasis was generally seen at the musculotendinous junction, during some resistance hip flexion or hyperextensive movements. Iliopsoas bursitis can be seen separately or in combination with strain. These events are usually seen together, and are the same in clinical manifestations (44). 

### 4.3. Interventions 

Tyler et al. (2001) were able to demonstrate that strengthening the adductor muscle group could be an effective method for preventing adductor sprains in professional ice hockey players (17). Considering incidence indicated by Lorentzon et al. who showed that the adductor strain is responsible for 10% of all hockey injuries (45), applying preventive measures could be potentially beneficial. In spite of the recognition of risk factors and augmenting intervention for hockey, adductor sprains continue to occur in the majority of exercises (45-47). Consequently, the high incidence of reoccurring cases may be inadequate rehabilitation or insufficient time for repair (41). Homlichet al. (1999) reported that an 8 to 12-week active intervention comprised of isometric adductional and abductional training, abdominal strengthening, balance training, and skate-like movements on a sliding sheet was more efficient in ameliorating chronic sprains, while passive physical therapy (e.g. massage, stretch, and other modalities) was not very effective for chronic type sprains (40). Tyler et al. (2002) also introduced a muscle injury plan for the injured throughout the phases of healing (41). 

In chronic conditions implementing a routine intervention of physical therapy may take up to 6 months to reach the optimum benefits. A well-known clinical trial in this field demonstrated that in returning chronic groin patients to their sport condition, active muscular training was more effective than passive physical therapy (40). Another prominent anatomical notion in groin pain is the locally distributed nerve fibers in the inguinal area. The ilio-inguinal and genital branch of the genito-femoral nerves, are both the most crucial sensory nerves, and to the greatest extent have the main role in non-acute forms of pain induced by groin injuries. Exercises of this type do need a robust eccentric contraction of adductors in sport activities (48). Adductor musculature strength has been related to the occurrence of some sprains. Careful management of injury and rehabilitative programs should be planned to minimize the hours of missing games while avoiding surgery (40). Adductor muscle sprains can result in considerable missed playing time for athletes in a variety of sports. Adductor sprains mostly occur in winter sports and football (3,7,35). A grading system presented by Lynch et al. (1999) is practical for prognosis and management (Table 3) (49). 

**Table 3.** Classification of Groin Sprains Prognosis Based on Muscle Tears (49).

Grading	Specifications
**Grade 1**	A minor tear with fiber damage of less than 10%
**Grade 2**	A moderate tear of fibers in the range of 10 to 90%
**Grade 3**	The most serious, either partial or full ruptures

According to this classification for groin sprains, adductor sprains are classified as a first-grade strain if there is pain with minimum loss of strength and mobilization. A second-grade strain is when there is an organic injury that suppresses the strength of the muscle while it does not include absolute loss of strength and motion. With the same rationale a third-grade strain is when there is an absolute collapse of the muscular/tendinous unit and complete loss of functional capabilities (49). Generally, in treatment issues it is worthy to note that implementing some passive physical therapy interventions (e.g. massaging, stretching) seems ineffective in the treatment of chronic groin sprains (12). Brukner et al. (2013) in a seven-point program refer to a treatment plan consisting rest, ice, compress and elevation (RICE) followed by soft tissue massage, stretching, core strengthening, progressive agility, neuromuscular control exercises, a graded running program and an isolated hamstring strengthening program with specific emphasis on eccentric exercises for the treatment of recurrent hamstring muscle injury (42). To summarize, rehabilitative interventions for the treatment of such injuries should minimize the lower extremities muscular imbalance and secure these structures over the energy-transferring phase of the exercise. Hence, with the implementation of an evidence-based rehabilitative program (22, 33, 50) the injured may return to his/her daily sport and limit further complications. 

## References

[1] Morelli V, Smith V (2001). Groin injuries in athletes.. Am Fam Physician..

[2] Macintyre J, Johson C, Schroeder EL (2006). Groin Pain in Athletes.. Curr Sport Med Rep..

[3] Ekstrand J, Gillquist J (1983). The avoidability of soccer injuries.. Int J Sports Med..

[4] Nicholas SJ, Tyler TF (2002). Adductor Muscle Strains in Sport.. Sport Med..

[5] Akita K, Niga S, Yamato Y, Muneta T, Sato T (1999). Anatomic basis of chronic groin pain with special reference to sports hernia.. Surg Radiol Anat..

[6] Albers SL, Spritzer CE, Garrett WE, Jr, Meyers WC (2001). MR findings in athletes with pubalgia.. Skeletal Radiol..

[7] Emery CA, Meeuwisse WH, Powell JW (1999). Groin and abdominal strain injuries in the National Hockey League.. Clin J Sport Med..

[8] Giza E, Mithofer K, Farrell L, Zarins B, Gill T (2005). Injuries in women's professional soccer.. Br J Sports Med..

[9] Knapik JJ, Bauman CL, Jones BH, Harris JM, Vaughan L (1991). Preseason strength and flexibility imbalances associated with athletic injuries in female collegiate athletes.. Am J Sports Med..

[10] Gomez E, DeLee JC, Farney WC (1996). Incidence of injury in Texas girls' high school basketball.. Am J Sports Med..

[11] Rao J, Zhou YX, Villar RN (2001). Injury to the ligamentum teres. Mechanism, findings, and results of treatment.. Clin Sports Med..

[12] Tyler TF, Silvers HJ, Gerhardt MB, Nicholas SJ (2010). Groin injuries in sports medicine.. Sports Health..

[13] Kluin J, den Hoed PT, van Linschoten R, I. Jzerman JC, van Steensel CJ (2004). Endoscopic evaluation and treatment of groin pain in the athlete.. Am J Sports Med..

[14] Molsa J, Airaksinen O, Nasman O, Torstila I (1997). Ice hockey injuries in Finland. A prospective epidemiologic study.. Am J Sports Med..

[15] Nielsen AB, Yde J (1989). Epidemiology and traumatology of injuries in soccer.. Am J Sports Med..

[16] Orchard J, Marsden J, Lord S, Garlick D (1997). Preseason hamstring muscle weakness associated with hamstring muscle injury in Australian footballers.. Am J Sports Med..

[17] Tyler TF, Nicholas SJ, Campbell RJ, McHugh MP (2001). The association of hip strength and flexibility with the incidence of adductor muscle strains in professional ice hockey players.. Am J Sports Med..

[18] Halabchi F, Ziaee V, Lotfian S (2007). Injury profile in women Shotokan karate championships in Iran (2004-2005).. J Sports Sci Med..

[19] Shadanfar K (2011). Sex-Related Injury Patterns among Iranian Professional Handball Players.. J Appl Environ Biol Sci..

[20] Arnason A, Sigurdsson SB, Gudmundsson A, Holme I, Engebretsen L, Bahr R (2004). Risk factors for injuries in football.. Am J Sports Med..

[21] Ekstrand J, Gillquist J (1983). Soccer injuries and their mechanisms: a prospective study.. Med Sci Sports Exerc..

[22] Hackney RG (2012). (iv) Groin pain in athletes.. Orthop Trauma..

[23] Freckleton G, Pizzari T (2013). Risk factors for hamstring muscle strain injury in sport: a systematic review and meta-analysis.. Br J Sports Med..

[24] Eirale C, Tol JL, Whiteley R, Chalabi H, Holmich P (2013). Different injury pattern in goalkeepers compared to field players: A three-year epidemiological study of professional football.. J Sci Med Sport..

[25] Ibrahim A, Murrell GA, Knapman P (2007). Adductor strain and hip range of movement in male professional soccer players.. J Orthop Surg (Hong Kong)..

[26] Renström PFH, Peterson L (1980). Groin injuries in athletes.. Br J Sport Med..

[27] Ekstrand J, Hilding J (1999). The incidence and differential diagnosis of acute groin injuries in male soccer players.. Scand J Med Sci Sports..

[28] Martens MA, Hansen L, Mulier JC (1987). Adductor tendinitis and musculus rectus abdominis tendopathy.. Am J Sports Med..

[29] Akermark C, Johansson C (1992). Tenotomy of the adductor longus tendon in the treatment of chronic groin pain in athletes.. Am J Sports Med..

[30] Kalebo P, Karlsson J, Sward L, Peterson L (1992). Ultrasonography of chronic tendon injuries in the groin.. Am J Sports Med..

[31] Maffey L, Emery C (2007). What are the risk factors for groin strain injury in sport? A systematic review of the literature.. Sports Medicine..

[32] Hoelmich P (1997). Adductor-related groin pain in athletes.. Sports Med Arthrosc..

[33] Holmich P, Holmich LR, Bjerg AM (2004). Clinical examination of athletes with groin pain: an intraobserver and interobserver reliability study.. Br J Sports Med..

[34] van Veen RN, de Baat P, Heijboer MP, Kazemier G, Punt BJ, Dwarkasing RS (2007). Successful endoscopic treatment of chronic groin pain in athletes.. Surg Endosc..

[35] Seward H, Orchard J, Hazard H, Collinson D (1993). Football injuries in Australia at the elite level.. Med J Aust..

[36] Emery CA, Meeuwisse WH (2001). Risk factors for groin injuries in hockey.. Med Sci Sports Exerc..

[37] Cowan SM, Schache AG, Brukner P, Bennell KL, Hodges PW, Coburn P (2004). Delayed onset of transversus abdominus in long-standing groin pain.. Med Sci Sports Exerc..

[38] Fricker PA, Taunton JE, Ammann W (1991). Osteitis pubis in athletes. Infection, inflammation or injury?. Sports Medicine..

[39] Morelli V, Espinoza L (2005). Groin injuries and groin pain in athletes: part 2.. Prim Care..

[40] Holmich P, Uhrskou P, Ulnits L, Kanstrup IL, Nielsen MB, Bjerg AM (1999). Effectiveness of active physical training as treatment for long-standing adductor-related groin pain in athletes: randomised trial.. Lancet..

[41] Tyler TF, Nicholas SJ, Campbell RJ, Donellan S, McHugh MP (2002). The effectiveness of a preseason exercise program to prevent adductor muscle strains in professional ice hockey players.. Am J Sports Med..

[42] Brukner P, Nealon A, Morgan C, Burgess D, Dunn A (2013). Recurrent hamstring muscle injury: applying the limited evidence in the professional football setting with a seven-point programme.. Br J Sports Med..

[43] Ekstrand J (1982). Soccer injuries and their prevention..

[44] Johnston CA, Wiley JP, Lindsay DM, Wiseman DA (1998). Iliopsoas bursitis and tendinitis. A review.. Sports Medicine..

[45] Lorentzon R, Wedren H, Pietila T (1988). Incidence, nature, and causes of ice hockey injuries. A three-year prospective study of a Swedish elite ice hockey team.. Am J Sports Med..

[46] Anderson K, Strickland SM, Warren R (2001). Hip and groin injuries in athletes.. Am J Sports Med..

[47] Hagglund M, Walden M, Ekstrand J (2006). Previous injury as a risk factor for injury in elite football: a prospective study over two consecutive seasons.. Br J Sports Med..

[48] Tegner Y, Lorentzon R (1991). Ice hockey injuries: incidence, nature and causes.. Br J Sports Med..

[49] Lynch SA, Renström PFH (1999). Groin Injuries in Sport.. Sport Med..

[50] Morelli V, Weaver V (2005). Groin injuries and groin pain in athletes: part 1.. Prim Care..

